# Treatment of Abscessed Teeth

**Published:** 1896-07

**Authors:** M. L. Rhein

**Affiliations:** New York.


ARTICLE VIII.

### TREATMENT OF ABSCESSED TEETH.

DR. M. L. RHEIN, NEW YORK.

Where the roots are straight, no better means of removing the contents of the diseased pulp-periphery of the root itself can be found than the Morey drill. The bad odor attached to the operation of drilling root-canals is due entirely to the attempted performance of this operation in roots where it is a mechanical impossibility. In such, having removed with the broach as much of the pulp as possible, we must then depend on chemical means. Two excellent agents have been introduced within the last few years—a combination of potassium and sodium and a 50 per cent solution of sulfuric acid. Either accomplishes the destruction of all the soft tissue leading to the very end of the root, and by either it will be found possible to reach the very end of every canal. The canals should then be thoroughly washed out by an injection of a powerful antiseptic solution, say a 1-500 solution of bichloride of mercury in hydrogen peroxid. This should be injected into the canals of the tooth with considerable force, by means of a hypodermic syringe with a platinum needle. Where pus has already begun to form, the forcing of a portion of this solution through the foramen of the root is productive of considerable pain to the patient, lasting sometimes, for one or two hours. On this account it has been generally condemned, and efforts made to cleanse the canal without using sufficient presssure to drive through the foramen any of the solution. This I believe to be a serious error. It is far preferable that the patient should suffer from the powerful effects of this antiseptic coagulating fluid on the tissues of the apical space, than to run the risk of leaving some minute portion of inlfitrated purulent matter, which might proceed to further development of an abscess. I have seen many alveolar abscesses aborted by this method of treatment, where considerable pus had already been evacuated. The canals should now be thoroughly dried, and a dressing of Ceylon cinnamon oil packed therein, hermetically sealed with gutta-percha, and allowed to remain till all symptoms of irritation have disappeared.

In other forms of acute abscess, where the infiltration of pus has already extended to some depth into the alveolar process, or where an external sinus has already been established, it is necessary, after the end of the root has been reached, to enlarge the opening at the apical point and thoroughly cauterize the tissue in the apical space by means of the same chemical agents used to reach the end of the root. Where the external sinus already exists, the injection of the bichloride solution should be so thorough that the fluid will make its exit through the external sinus.

Beware of over-treatment. At the very same sitting, the canal having been thoroughly dried, the end of the root should at once be hermetically sealed by chloro-percha, and the patient instructed in the use of a proper mouth-wash. No further operative interference ought to be necessary.

Where, however, no external opening has been obtained, and we have infiltration of pus extending to the alveolar tissue, it is necessary to move with more caution. Make a similar dressing of cinnamon oil packed in the roots, and renew this once or twice, till certain that every vestige of the purulent infiltration has been destroyed—*Cosmos.*

